# Light-Mediated Population Dynamics of Picocyanobacteria Shaping the Diurnal Patterns of Microbial Communities in an Atoll Lagoon

**DOI:** 10.3390/microorganisms13040727

**Published:** 2025-03-24

**Authors:** Ying Yu, Maosen Shangguan, Ping Sun, Xiaofeng Lin, Jiqiu Li

**Affiliations:** 1 Key Laboratory of the Ministry of Education for Coastal and Wetland Ecosystem, The Fujian Provincial Key Laboratory for Coastal Ecology and Environmental Studies, College of the Environment and Ecology, Xiamen University, Xiamen 361102, China; yuying719@126.com (Y.Y.); psun@xmu.edu.cn (P.S.); linxf@xmu.edu.cn (X.L.); 2 Nansha Islands Coral Reef Ecosystem National Observation and Research Station, Guangzhou 510300, China; sgms@scs.mnr.gov.cn; 3 South China Sea Environmental Monitoring Center, State Oceanic Administration, Guangzhou 510300, China; 4 State Key Laboratory of Marine Environmental Science, Xiamen University, Xiamen 361102, China

**Keywords:** atoll lagoon, diurnal patterns, flow cytometry, high-throughput sequencing, light, microbial community, picocyanobacteria

## Abstract

The diurnal cycle of light significantly impacts microbes, making diurnal investigations crucial for understanding microbial communities. Zhubi Reef is known to harbor exceptionally rich biodiversity, with both zooplankton and seawater properties demonstrating diurnal patterns. However, microbial community structures and their potential diurnal dynamics remain largely unexplored. This study is the first to utilize flow cytometry and high-throughput sequencing to investigate prokaryotic and microeukaryotic communities in the Zhubi lagoon, focusing on diurnal variations under different light intensities. The picophytoplankton cell abundance and the microbial community structures both exhibit clear diurnal variations. Light is identified as the primary driver of diurnal variations in the picophytoplankton cell abundance. The diurnal variation in microbial community diversity is driven by changes in the cell abundance of two dominant picocyanobacterial groups. Our findings reveal the diurnal variation in microbial community structures is mediated by the light-driven fluctuation of dominant cyanobacterial populations, and the diurnal variation patterns of specific populations may vary with habitats and sampling timepoints. This research provides valuable insights into the microbial community structure within the Zhubi lagoon.

## 1. Introduction

Marine planktonic microorganisms constitute approximately 60% of the total ocean biomass [1]. Among these microorganisms, the dynamics of key taxa drive the primary productivity and energy flow efficiency in the ocean, regulate the microbial community structure, and even drive globally important biogeochemical cycles [2,3,4,5]. Coral reefs are often regarded as oases of biodiversity and productivity in the oligotrophic open ocean [6,7,8]. Their high productivity largely depends on the capture and recycling of nutrients by reef-associated microbial communities, which are fundamental drivers of biogeochemical cycling in coral reef waters [9,10,11].

Numerous studies have investigated marine microbial communities on the diurnal scale. The preferences of different microbial groups for specific environmental conditions, coupled with their potential interactions, give rise to distinct diurnal dynamics within these communities [12,13,14,15]. These findings provide a comprehensive diurnal-scale understanding of microbial communities and functional processes in coral reef ecosystems. Microorganisms in seawater from the coral reef exhibit significant temporal fluctuations [16]. However, microbial community diurnal patterns may not be always observed in all studies. Some studies report significant diel variations in coral reef seawater microbial communities [17,18]; some even exceed those caused by geographic differences [19]. In contrast, research also indicates that these communities exhibit no diel fluctuations [20]. During the diurnal cycle, light undergoes periodic light–dark variations, directly influencing the physiology and functions of aquatic organisms [21,22]. Light drives a range of diurnal dynamics in microbial processes, including photoproduction [23], zooplankton grazing [24], viral lysis [25], and heterotrophic bacterial metabolism [26], which implies its potential capability triggering diurnal community dynamics. Notably, several studies of coral reef seawater diurnal microbial dynamics attribute these variations primarily to dominant taxa abundances, such as *Psychrobacter* sp. [19] and picocyanobacteria [18], rather than the direct light impact. The population dynamics of light-sensitive species are strongly influenced by variations in light intensity. These abundance variations can be amplified or neutralized to the whole community through ecological mechanisms such as competition and mutualism [27,28].

Zhubi Reef, located in the northern part of the Nansha Islands, is a typical circular coral atoll with an inner lagoon. Due to its favorable geographical location and distinctive topographical features, previous surveys of fish, phytoplankton, and zooplankton have consistently highlighted the lagoon’s exceptional biodiversity [29,30,31]. However, as key components of the microbial food web, the diversity of prokaryotes and microeukaryotes in the Zhubi lagoon remains poorly understood. Furthermore, only two studies have conducted diurnal sampling of seawater in the Zhubi lagoon, revealing distinct diurnal patterns in primary production, physicochemical properties, and zooplankton diel vertical migration [32,33]. Based on these findings, it is likely that similar diurnal dynamics also occur within the microbial communities.

In this study, we investigate the microbial communities in the Zhubi lagoon, focusing on the diurnal dynamics of picoplankton cell abundance and microbial community structures in relation to environmental factors. We hypothesize that (1) diurnal light variations are critical to dominant taxa dynamics, and (2) microbial communities experience significant changes in diversity and composition during the diurnal cycle, driven by dominant taxa abundances.

## 2. Materials and Methods

### 2.1. Sampling Site

From April to June 2023, the survey was conducted aboard the Xiangyanghong 14 within the Nansha Islands. The investigated Zhubi Reef (10°55′ N, 114°05′ E) is one of the typical small, enclosed atolls in the Nansha Islands, with a lagoon covering approximately 9.5 km ^2^ and a maximum depth less than 25 m [33]. The lagoon is encircled by reef barriers, reducing wave energy, calming the lagoon waters, and creating a shelter for multifarious plankton [29,32,34]. Only at high tide is the reef submerged, allowing for the exchange of water between the open sea and the lagoon.

**Figure 1:**
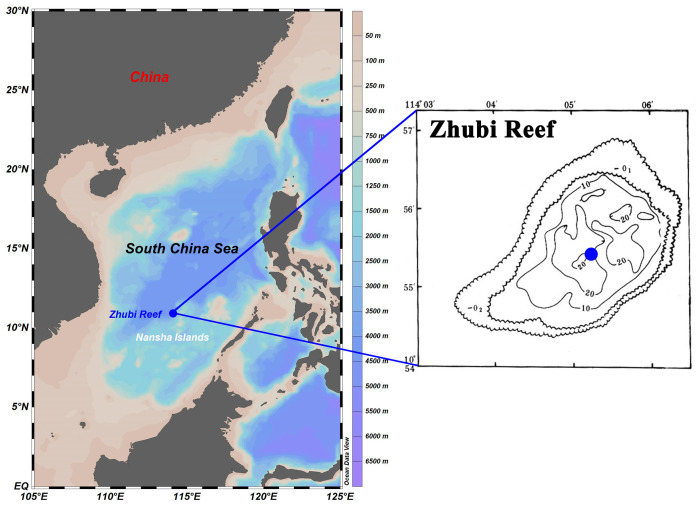
Location and bathymetric map of the Zhubi Reef. Blue dots indicate the specific sampling sites. Sampling site maps were generated using Ocean Data View version 5.6.3 (http://odv.awi.de, accessed on 15 October 2022). The bathymetric map was sourced from the South China Sea Ocean Data Center.

The water samples were taken with an organic glass hydrophore at the deepest point of the lagoon (Figure 1). Water samples were collected at 08:30, 13:30, and 20:30 with different light intensities. Furthermore, seawater was collected at three depths (1 m, 10 m, and 20 m). Sampling was performed over consecutive days to ensure that each timepoint was sampled three times, with no extreme weather events occurring during the sampling period. In summary, a total of 27 water samples were collected. Details of sample information are shown in Table S1.

### 2.2. Measurement of Environmental Variables

During the collection of water samples, photosynthetically active radiation (PAR) was measured at different depths in situ using a Compact Optical Profiling System (C-OPS) (Biospherical Instruments Inc., San Diego, CA, USA). Depth, temperature, salinity, and dissolved oxygen (DO) concentrations were measured in situ with a YSI EXO2 multiparameter water quality sonde (YSI Inc., Yellow Springs, OH, USA). Tidal height data were obtained from the National Marine Data and Information Service. Water samples were collected for chlorophyll *a* concentration analysis by filtering through GF/F membranes (47 mm diameter, Whatman, Cytiva, UK) and stored at −20 °C until processed. Chlorophyll *a* was extracted with acetone and measured using a Trilogy fluorometer (Turner Designs, San Jose, CA, USA) [35]. Water samples for nutrient analysis were filtered through a 0.45 μm hydrophilic filter (Millipore, Billerica, MA, USA) and stored in polypropylene (PP) containers at −20 °C, with chloroform added to a final concentration of 0.2%. The concentrations of nitrite (${\mathrm{NO}}_{2}^{-}$), dissolved silicate (DSi), and soluble reactive phosphate (SRP) were analyzed using a continuous flow analyzer (Seal AA3, Norderstedt, Germany). Abundance samples of picoplankton (*Prochlorococcus*, *Synechococcus*, pico-sized pigmented eukaryotes, and heterotrophic bacteria) were filtered through 20 μm mesh into two 2 mL tubes, then fixed with glutaraldehyde (1% final concentration) for 15 min in the dark before freezing at −20 °C. To enumerate bacterial abundance, one sample was stained with SYBR Green I nucleic acid gel stain (final concentration 1:10,000; Invitrogen, Carlsbad, CA, USA) and incubated in the dark for 15 min, then analyzed using a CytoFLEX flow cytometer (Beckman Coulter, Brea, CA, USA) following the protocol described by Marie et al. (2001) [36]. The other sample was directly analyzed using the CytoFLEX flow cytometer (Beckman Coulter, Brea, CA, USA) for the quantification of picophytoplankton populations, including *Prochlorococcus*, *Synechococcus*, and pico-sized pigmented eukaryotes.

### 2.3. DNA Sampling, Extraction, Processing, and Amplicon Sequencing

The microbial community was collected by filtering 2 L of seawater onto a 0.22 μm pore-sized polycarbonate membrane (47 mm diameter, Millipore, USA). Filters were immersed in Sample Protector for RNA/DNA (Takara Bio, Tokyo, Japan) at room temperature for 1 h and subsequently frozen at −20 °C until further processing. Total DNA was extracted from the polycarbonate membrane using the DNeasy PowerWater^®^ Kit (Qiagen, Hilden, Germany), following the manufacturer’s instructions, and then sent for library construction and high-throughput sequencing at GENEWIZ (Suzhou, China). The sequencing library was constructed using a MetaVX Library Preparation Kit (GENEWIZ, Suzhou, China). The amplicons covering the V4 hypervariable region of the prokaryotic 16S rRNA gene and the eukaryotic 18S rRNA gene were generated using a bacterium-specific primer set (515F and 806R) [37] and a eukaryote-specific primer set (528F and 706R) [38], respectively. The PCR conditions followed the default parameters provided by the GENEWIZ company. Finally, the paired-end (PE) sequencing was carried out with the Illumina Miseq Platform (Illumina Inc., San Diego, CA, USA) at Genewiz, Inc. Quality control for the sequencing libraries was also carried out by GENEWIZ company.

### 2.4. Sequence Data Processing

Sequencing data were processed using the QIIME2 v2019.4 pipeline [39]. Quality control and denoising were performed with DADA2 to obtain the amplified sequence variant (ASV), and a dataset of representative sequences was taken for subsequent analysis [40]. The 16S rRNA and 18S rRNA gene representative sequences of ASVs were assigned to prokaryotic and microeukaryotic taxa using the RDP Classifier (Ribosomal Database Program) Bayez algorithm [41], based on the Silva 138 database [42] and Protist Ribosomal Reference (PR2) database [43], respectively. The ASVs classified as Archaea, chloroplasts, and mitochondria, or those not attributed to bacteria, were excluded from the prokaryotic dataset. Likewise, ASVs identified as macroorganisms, including Metazoa and higher plants (Rhodophyta, Streptophyta, and Ulvophyceae), were removed to focus exclusively on single-celled microeukaryote. Prokaryotic and microeukaryotic ASV tables were rarefied to the minimum of the sequencing depth (71,294 and 26,397 sequences per sample, respectively) to avoid potential biases from uneven sequencing depths.

### 2.5. Statistical Analysis

Most statistical analyses were performed using the “vegan” package [44] in R version 4.3.1 [45]. Alpha diversity was assessed using four indices, including ASV richness, Shannon, evenness, and Faith’s phylogenetic diversity (PD). Differences in environmental variables, picophytoplankton abundances, and alpha diversity indices under different light conditions during the diurnal cycle were examined using the Wilcoxon rank sum test. The “MASS” package was used to perform stepwise regression modeling to analyze the relative contributions of environmental variables to variations in picophytoplankton abundances and alpha diversity indices of prokaryotic and microeukaryotic communities. An analysis of similarities (ANOSIM) was performed to test the effects of diurnal light intensity and depth on the community structure of prokaryotes and microeukaryotes. Sample dendrograms were constructed using the unweighted pair group method, with arithmetic mean (UPGMA) hierarchical clustering based on Bray–Curtis distance analyses. Non-metric multidimensional scaling (NMDS) analysis based on Bray–Curtis dissimilarity was performed to visualize community dissimilarities. The Mantel test based on Spearman’s correlation was used to unveil the correlations between potential drivers (biotic and environmental variables) and community dissimilarity based on Bray–Curtis distance using the “linkET” package. Significance was assessed using 999 permutations. To develop a more holistic understanding of the direct and indirect effects of light and picocyanobacteria on the diversity (both alpha and beta) of prokaryotic and microeukaryotic communities, we used the “plspm” package to perform partial least squares path modeling (PLS-PM) [46].

## 3. Results

### 3.1. Variations in the Environmental Conditions of Lagoon Seawater

During the sampling period, the temperature of lagoon seawater in Zhubi Reef ranged from 29.50 °C to 29.71 °C, salinity from 34.73 to 34.80 PSU, dissolved oxygen from 5.97 to 6.29 mg/L, and chlorophyll *a* concentration from 0.35 to 0.81 μg/L. For nutrients, DSi ranged from 2.76 to 3.13 μmol/L, while SRP and nitrite levels were extremely low, with some SRP levels even falling below the detection limit. The DSi:SRP ratios exceeded 60:1 in all samples, indicating strong P limitation of phytoplankton communities. Noon is generally close to the high tide, while the low tide occurs at night (Figure S1). Only temperature and chlorophyll *a* exhibited significant diurnal variations, with morning temperatures significantly lower than those at noon and in the evening, while the morning chlorophyll *a* concentration was significantly higher than that in the evening (Figure S2). However, dissolved oxygen and salinity exhibited significant depth-related variations, with salinity significantly lower and dissolved oxygen significantly higher in shallow layers (Figure S2).

Under illuminated conditions, PAR attenuated rapidly within the water column (Figure S3). At a depth of 20 m, the light intensity is approximately 10% of that at 1 m. At noon, the light intensity at 1 m can reach 2057.2 μmol photons m^−2^ s^−1^, roughly twice the value observed at the same depth in the morning. Variations in cloud cover across sampling days led to slight fluctuations in light intensity at the same depth.

### 3.2. Picoplankton Cell Abundance

**Figure 2:**
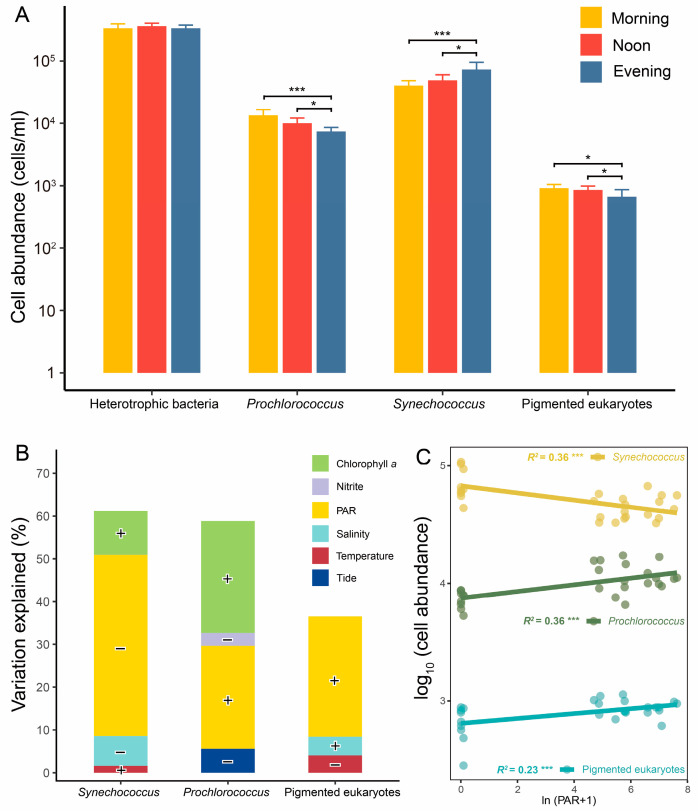
Cell abundance of picoplankton and its regressions and correlations with environmental variables. Comparison of picoplankton cell abundance among different times (**A**). Regressions (**B**) and correlations (**C**) between cell abundances of picophytoplankton and environmental variables. *, *p* < 0.05; ***, *p* < 0.001. The contributions of selected environmental variables were determined using stepwise regression models. Positive correlations between environmental variables and cell abundances are denoted by “+”, while negative correlations are indicated by “−”.

Heterotrophic bacterial cell abundances ranged from 2.7 to 4.5 × 10^5^ cells/mL, with no significant diurnal variation (Figure 2A). Among the three picophytoplankton groups, *Synechococcus* was the dominant taxon, with abundances ranging from 3.3 to 10.8 × 10⁴ cells/mL. The three picophytoplankton groups exhibited significant differences in cell abundances between the light conditions (morning, noon) and dark condition (evening) (Figure 2A). Under light conditions, the cell abundances of *Prochlorococcus* and pico-sized pigmented eukaryotes were significantly higher than under dark conditions, whereas *Synechococcus* exhibited an opposite pattern. However, depth did not cause significant differences in any picoplankton cell abundances (Figure S4).

According to stepwise regression models, PAR intensity was identified as the most significant factor influencing the diurnal variations in picophytoplankton cell abundances (Figure 2B and Table S2). It explained 26.7%, 49.2%, and 26.3% of the variations in the cell abundances of *Prochlorococcus*, *Synechococcus*, and pico-sized pigmented eukaryotes, respectively. Notably, PAR intensity showed a negative correlation with cell abundances of *Synechococcus*, while the other two types of picophytoplankton displayed positive correlations (Figure 2C). Additionally, chlorophyll *a* exhibited a significant positive correlation with the cell abundance of *Synechococcus* and *Prochlorococcus* (Figure 2B and Table S2).

### 3.3. Microbial Community Diversity in the Lagoon

**Figure 3:**
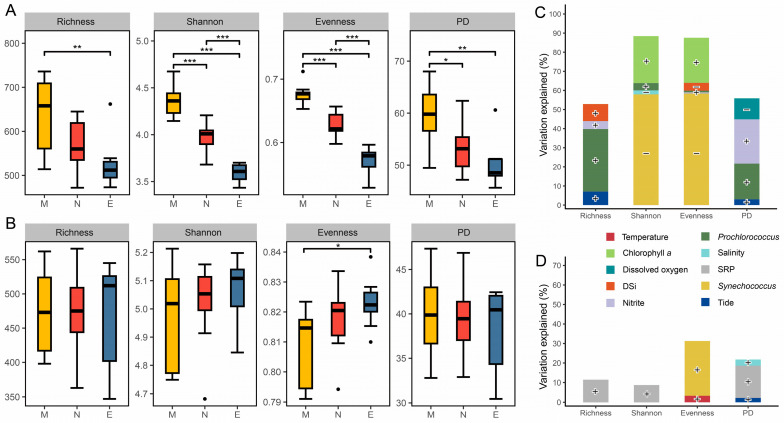
Alpha diversity of microbial communities and environmental factors explaining its variations. Boxplots illustrate the comparison of alpha diversity indices for prokaryotic (**A**) and microeukaryotic (**B**) communities among times. Relative contributions of environmental and biotic variables in explaining the variations in alpha diversity indices of prokaryotic (**C**) and microeukaryotic (**D**) communities based on stepwise regression models. M, morning; N, noon; E, evening; DSi, dissolved silicate; SRP, soluble reactive phosphate. *, *p* < 0.05; **, *p* < 0.01; ***, *p* < 0.001 (Wilcoxon rank sum test). “•” Indicates greater than 1.5 times of the interquartile range. Positive correlations are denoted by “+”, and negative correlations by “−”.

After quality filtering, sequencing of 27 samples yielded 1,924,938 high-quality tags, which clustered into 5397 prokaryotic ASVs, and 712,719 high-quality tags, which clustered into 5252 microeukaryotic ASVs. Analysis of four types of alpha diversity indices (ASV richness, Shannon, evenness, and PD) for the prokaryotic community revealed significant diurnal variation across different times, whereas the microeukaryotic community showed significant differences only in evenness between morning and night (Figure 3A,B). All alpha diversity indices of prokaryotes were significantly higher under light conditions (morning, noon) than under dark conditions (evening), with morning values being significantly higher than those at noon (Figure 3A). The evenness of microeukaryotes was significantly higher at night compared to the morning (Figure 3B). Regarding depth, the alpha diversity indices of prokaryotes and microeukaryotes did not show significant differences (Table S3). However, significant differences in the individual alpha diversity indices of prokaryotes between depths were observed only when the samples were separated by time (*n* = 9), specifically, at noon and night.

Stepwise regression models indicate that the contributions of biotic variables to the alpha diversity indices of both prokaryotes and microeukaryotes outweighed those of environmental variables (Figure 3C,D and Table S4). The richness and PD indices of the prokaryotic community were positively correlated with the cell abundance of *Prochlorococcus*, explaining 32.7% and 18.7% of its variation, respectively. In contrast, the Shannon and evenness indices were negatively correlated with the cell abundance of *Synechococcus*, accounting for 57.9% and 58.3% of its variation. Additionally, chlorophyll *a* concentration was positively correlated with both the Shannon and evenness indices, explaining 24.6% and 23.6% of their variation. Among the abiotic factors, DSi and dissolved oxygen were negatively correlated with the evenness and PD indices (Figure 3C and Table S4). For the alpha diversity indices of microeukaryotic communities, the stepwise regression models showed generally poor explanatory power (Figure 3D and Table S4). Variations in richness and PD indices were significantly positively correlated with SRP, accounting for only 11.4% and 16.4% of the variation, respectively. The sole index exhibiting diurnal differences, evenness, was significantly positively correlated with the cell abundance of *Synechococcus*, explaining 27.9% of its variation.

### 3.4. Community Structure of Microbes in the Lagoon

**Figure 4:**
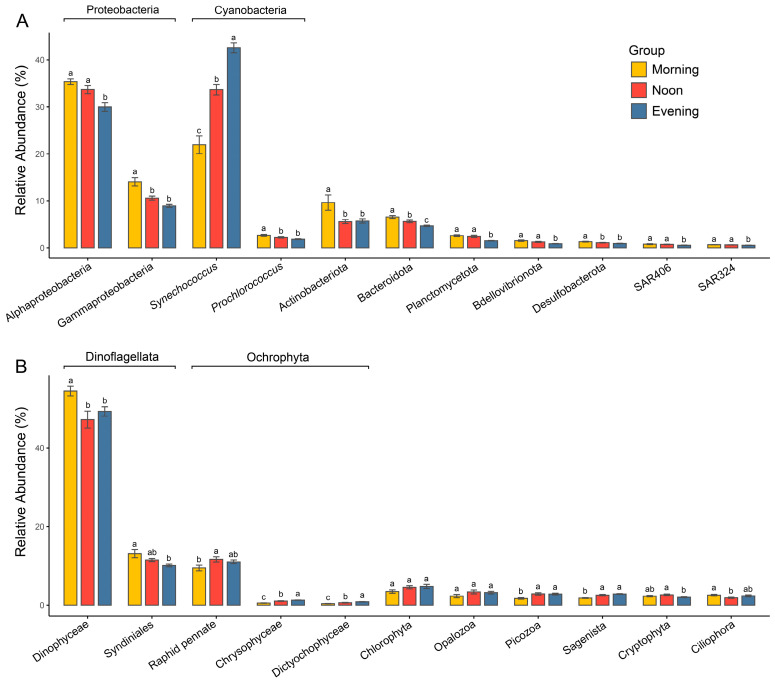
Comparisons of relative abundances of major prokaryotic (**A**) and microeukaryotic (**B**) lineages among different times. Different letters represent significant difference (*p* < 0.05, one-way ANOVA).

The prokaryotic communities in the Zhubi lagoon are primarily composed of Proteobacteria (predominantly Alphaproteobacteria and Gammaproteobacteria) and Cyanobacteria (almost exclusively represented by the genera *Synechococcus* and *Prochlorococcus*), contributing 44.2% and 35.1% to the total relative abundance, respectively (Figure 4A and Figure S5A). Actinobacteriota and Bacteroidota contribute 7% and 5.6% to the total relative abundance of the community, respectively. Other groups, including Planctomycetota, Bdellovibrionota, Desulfobacterota, and the SAR406 and SAR324 clades, constitute minor components of the prokaryotic community. The relative abundances of these major prokaryotic groups varied significantly across times (Figure 4A). Significant differences in the relative abundance of *Synechococcus* were observed across the three timepoints (21.9% vs. 33.7% vs. 42.6%), with its highest abundance recorded in the evening. In contrast, the relative abundances of all other groups were significantly higher in the morning than in the evening. The relative abundances of Alphaproteobacteria, Bacteroidota, Planctomycetota, Bdellovibrionota, the SAR406 clade, and the SAR324 clade were significantly higher at noon compared to the evening. Meanwhile, the relative abundances of Gammaproteobacteria, *Prochlorococcus*, Actinobacteriota, Bacteroidota, and Desulfobacterota were significantly higher in the morning than at noon.

The microeukaryotic community was overwhelmingly dominated by Dinoflagellata, which accounted for about 62% of the total relative abundance (Figure 4B and Figure S5B). This was followed by Ochrophyta at 13.8% and Chlorophyta at 4.3%. Other groups, such as Opalozoa, Picozoa, Sagenista, Cryptophyta, and Ciliophora, constituted only a minor proportion of the microeukaryotic community. The relative abundance of different microeukaryotic taxa exhibited inconsistent variation trends among different times (Figure 4B). Dinoflagellata exhibited significantly higher relative abundance in the morning compared to the evening, while the primary group of Ochrophyta, Raphid pennate, showed a significantly lower relative abundance in the morning than at noon, with no significant difference between morning and evening. Additionally, the relative abundance of Chlorophyta did not differ significantly across timepoints.

**Figure 5:**
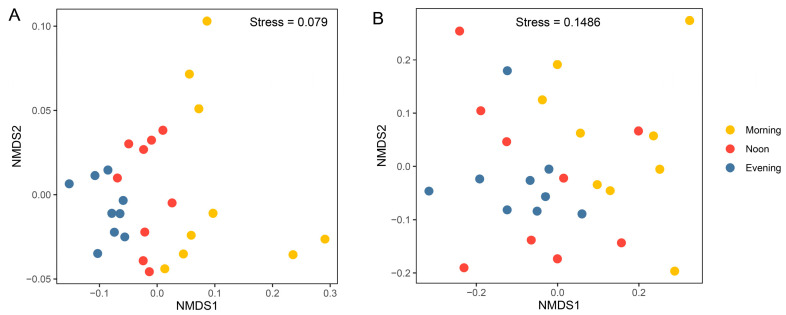
Non-metric multidimensional scaling (NMDS) ordinations of prokaryotic (**A**) and microeukaryotic (**B**) communities, based on the Bray–Curtis distance for all samples (*n* = 27).

Results from the nMDS analysis based on Bray–Curtis distances showed significant separation of prokaryotic community samples among different times (Figure 5A; *R* = 0.53, *p* < 0.001, ANOSIM test; Table 1), suggesting that significant diurnal variations occurred in the prokaryotic community. Even when considering samples from each depth separately, the influence of temporal variation remains evident (1 m: *R* = 0.53, *p* < 0.01; 10 m: *R* = 0.32, *p* < 0.05; 20 m: *R* = 0.51, *p* < 0.01; Table 1). For the microeukaryotic community, the NMDS results show a clear separation between the morning and evening samples (Figure 5B; *R* = 0.21, *p* < 0.001; Table 1). When considering each depth separately, the influence of temporal variation is only significant at 1 m (*R* = 0.41, *p* < 0.05; Table 1). Dendrograms generated by the unweighted pair group method with arithmetic mean (UPGMA) clustering exhibited a consistent pattern (Figure S5).

### 3.5. Environmental Drivers of Microbial Community in the Lagoon

**Figure 6:**
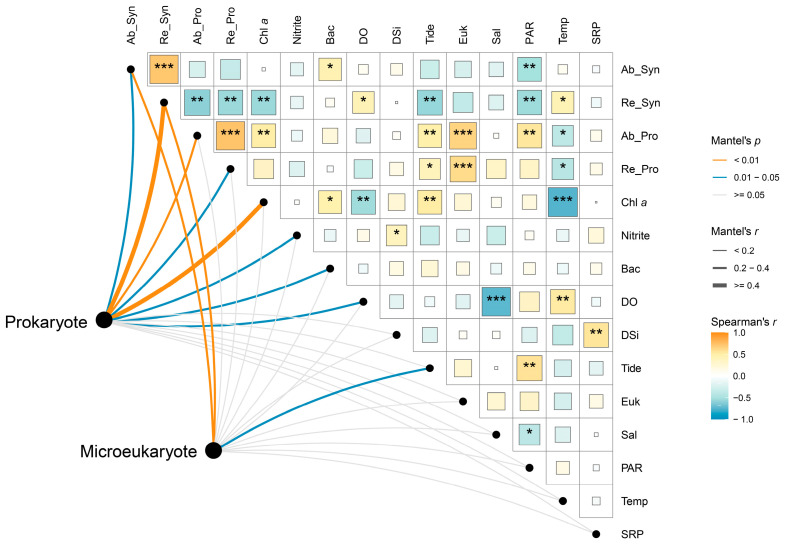
Spearman correlations and Mantel test reveal environmental–microbial relationships. The lines connecting environmental variables and microbial communities represent the strength of their relationship. A larger line indicates a stronger relationship. The color of the blocks and the asterisks reflects the relationships between environmental variables. Ab_Syn, cell abundance of *Synechococcus*; Re_Syn, relative abundance of *Synechococcus*; Ab_Pro, cell abundance of *Prochlorococcus*; Re_Pro, relative abundance of *Prochlorococcus*; Chl *a*, chlorophyll *a* concentration; Bac, cell abundance of heterotrophic bacteria; DO, dissolved oxygen; Tide, tidal height; Euk, cell abundance of pico-sized pigmented eukaryotes; Sal, salinity; Temp, temperature. *, *p* < 0.05; **, *p* < 0.01; ***, *p* < 0.001.

The Mantel test results indicated that the prokaryotic community was more strongly influenced by environmental variables than the microeukaryotic community (Figure 6). Among the environmental variables considered, both the absolute and relative abundances of *Synechococcus* were identified as the most influential factors for both the prokaryotic (absolute: *R* = 0.21, *p* < 0.05; relative: *R* = 0.66, *p* < 0.001; Table S5) and microeukaryotic (absolute: *R* = 0.26, *p* < 0.01; relative: *R* = 0.31, *p* < 0.01; Table S5) communities (Figure 6). In addition, the prokaryotic community was significantly influenced by the absolute and relative abundances of *Prochlorococcus*, chlorophyll *a* concentration, and the cell abundance of heterotrophic bacteria. Among the abiotic factors, nitrite and dissolved oxygen had a significant impact on the prokaryotic community, while tidal height significantly affected the microeukaryotic community.

**Figure 7:**
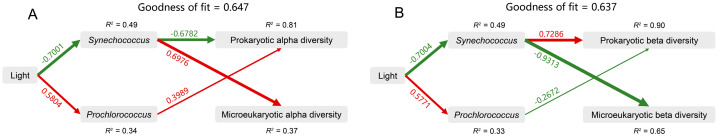
The partial least squares path model (PLS-PM) showing the direct and indirect effects of light, *Synechococcus*, and *Prochlorococcus* on the alpha diversity (**A**) and beta diversity (**B**) of prokaryotic and microeukaryotic communities. The width of the arrow is proportional to the strength of the path coefficient. The red and green arrows indicate the positive and negative flow of causality, respectively (*p* < 0.05). The number on the arrow indicates the effective normalized path coefficient. R^2^ represents the variance of the dependent variable explained by the model. *Synechococcus*, the absolute and relative abundance of *Synechococcus*; *Prochlorococcus*, the absolute and relative abundance of *Prochlorococcus*.

Furthermore, we used PLS-PM to analyze the direct and indirect effects of light and the abundance (absolute and relative) of *Synechococcus* and *Prochlorococcus* on the microbial alpha and beta diversities (Figure 7A,B). For microbial alpha diversity, the model fit the data well, with a goodness of fit of 0.647 (Figure 7A). Light had the greatest total positive impact on prokaryotic alpha diversity (0.697), and the abundance of *Synechococcus* had the greatest total positive impact on microeukaryotic alpha diversity (0.698) (Figure S6A,B). For microbial beta diversity, the model fit the data well, with a goodness of fit of 0.637 (Figure 7B). The abundance of *Synechococcus* had the greatest total positive impact on prokaryotic beta diversity (0.767) and the greatest total negative impact on microeukaryotic beta diversity (−0.931) (Figure S6C,D). These results suggest that light intensity and the abundance of *Synechococcus* were the key drivers of variations in microbial community diversity within the Zhubi lagoon.

## 4. Discussion

### 4.1. Light as the Key Factor Shaping Lagoon Picophytoplankton Cell Abundance

The stepwise regression model revealed that light was a key factor in explaining the diurnal variations in cell abundances of all three types of picophytoplankton. Phytoplankton abundance results from a dynamic balance between gain and loss processes. Cell division represents the primary gain process, while loss processes include viral lysis, light stress, grazing, and other factors [47]. However, our study focused specifically on light intensity and did not quantify other factors. Previous studies have shown that the diurnal variations of other factors are largely directly or indirectly driven by light [24,25,48]. Therefore, the diurnal light cycle plays a crucial role in diurnal picophytoplankton cell abundance fluctuations.

Similarly, a study conducted in the lagoon of Ahe Atoll (French Polynesian Tuamotu Archipelago) reported similar findings that the diurnal cell abundance variations of three picophytoplankton types were significantly negatively correlated with light [48]. In general, phytoplankton perform photosynthesis and growth during the day and divide at night [48]. Therefore, in continuous time-series sampling studies covering the diurnal cycle, picophytoplankton abundance typically peaks at night, with a trough observed during the day [49,50,51]. However, in our study, only *Synechococcus* cell abundance showed a negative correlation with light, whereas *Prochlorococcus* and pico-sized pigmented eukaryotes exhibited a positive correlation. It is particularly unexpected that the cell abundance peaks of *Prochlorococcus* occurred during the day, as it is highly sensitive to solar radiation and generally prefers low-light environments in the open ocean [52,53]. Although counterintuitive, this result is not unique. A study on the Yongle Blue Hole also recorded a daytime peak in *Prochlorococcus* abundance in the surface layer, with the nighttime peak observed at a depth of 40 m [54]. Similarly, Ribalet et al. (2015) reported a daytime increase in *Prochlorococcus* abundance and a decrease at night across two separate cruises [55]. In fact, the division times of different strains within the same phytoplankton group may vary [56]. In addition to being influenced by genetic factors, light intensity and nutrient conditions also affect the cell division phase, resulting in different diurnal patterns [57,58]. Given the unique habitat characteristics of atoll lagoons, it is plausible that certain picophytoplankton strains may emerge differing from those in other environments. However, since no continuous time-series observations have been conducted, the peak abundance times for each picophytoplankton group in the Zhubi lagoon remains unclear. Still, Zhubi picophytoplankton exhibit a distinct diurnal pattern in abundance, and their peak time varies among types. In addition, the similar patterns in *Prochlorococcus* and pico-sized pigmented eukaryotes cell abundances may suggest that their peak cell abundances occur spontaneously and that both are regulated by similar grazing controls.

### 4.2. Dominant Picocyanobacteria Driving Lagoon Microbial Diversities

Consistent with other atoll lagoon reports, the picophytoplankton community in the Zhubi lagoon was dominated by *Synechococcus*, which was attributed to the light inhibition and nutrient characteristics of shallow lagoons [59,60,61,62]. We found that biotic factors explained the variations in the alpha and beta diversity of both the prokaryotic and microeukaryotic communities across diurnal light variations in the Zhubi lagoon more effectively than environmental variables. Moreover, the explanation can largely be attributed to the abundance of *Synechococcus*. Although *Synechococcus* is only slightly larger than bacteria in cell size, 40% of *Synechococcus* in natural environments have bacteria attached to their cell surface [63]. These cyanobacteria can serve as diverse carbon sources, closely interacting with prokaryotes and significantly influencing their community structure [64,65]. In coral reef habitats where *Synechococcus* occupies a considerable proportion, it often has a substantial impact on prokaryotic diversity [18,66]. In our study, both the stepwise regression model and PLS-PM reveal that *Synechococcus* abundance has a negative impact on prokaryotic alpha diversity. An increase in the *Synechococcus* population abundance leads to a decrease in community alpha diversity, for it occupies a larger share of the prokaryotic community. However, according to Weber and Apprill (2020), even after excluding the *Synechococcus* ASVs from the diversity analysis, this downward trend remained unchanged, indicating that the diversity changes were observed across the entire community, rather than being limited to dominant picocyanobacterial populations [18]. Additionally, studies have shown that the abundances of *Synechococcus* and certain bacterial groups are regulated by the phosphate concentration [67,68]. Given the extremely low phosphate levels detected in the Zhubi lagoon seawater, this negative correlation suggests potential competition for phosphate among prokaryotic groups. For the prokaryotic beta diversity, the PLS-PM analysis shows that *Synechococcus* abundance has a significant positive impact. Studies have indicated that different *Synechococcus* strains may support completely distinct prokaryotic communities. When bacteria from the same seawater are co-cultured with different *Synechococcus* strains, only about 40% of the species are shared between the two resulting prokaryotic communities [64]. This suggests that as *Synechococcus* abundance increases, different strains may support more diverse prokaryotic communities.

In addition, *Synechococcus* abundance was also significantly correlated with the microeukaryotic diversities. According to the PLS-PM analysis, the impact of *Synechococcus* abundance on microeukaryotic diversities is opposite to the prokaryotic results: positive effect on alpha diversity while negative on beta diversity. Microeukaryotes are typically grazers of *Synechococcus*, including nanoflagellates and ciliates [69,70,71,72]. Studies have shown that grazers exhibit condition-dependent feeding on *Synechococcus*. For example, nanoflagellates exhibit size preferences for *Synechococcus*, leading them to feed more actively during the night when division occurs [73]. Additionally, Strom (1991) demonstrated that *Gymnodinium*-like dinoflagellates engage in significant ingestion only when the prey density exceeds 20 µg C L^−1^, suggesting a grazing threshold for *Synechococcus* [74]. Similarly, *Tetraselmis* species have been observed to ingest *Synechococcus* when its density is high [75]. Given that we observed the highest abundance of *Synechococcus* at night, this could support the proliferation of specific grazers, thereby contributing to a more homogenized community structure.

A substantial portion of the variation in prokaryotic alpha diversity is explained by *Prochlorococcus*, with a significant positive correlation. According to PLS-PM, an increase in *Prochlorococcus* abundance has a certain negative impact on prokaryotic beta diversity. Some studies suggest that microbial signatures characterized by high alpha diversity, high community similarity, and a high prevalence of *Prochlorococcus* can serve as key indicators of a healthy coral reef, indicating that a positive correlation among the three exists under certain conditions [76]. On the other hand, the positive correlation between *Prochlorococcus* abundance and prokaryotic alpha diversity actually reflects the negative correlation between the abundances of the two picocyanobacteria, a relationship that has often been documented [77,78].

### 4.3. Diurnal Variation in the Relative Abundance of Prokaryotic and Microeukaryotic Populations

In this study, we find that Proteobacteria and Cyanobacteria dominated the prokaryotic community, while Dinoflagellata dominated the microeukaryotic community, across all samples, consistent with previous reports on coral reef environments [79,80,81,82,83,84]. Additionally, the proportions and variation trends of the two types of picocyanobacteria abundances obtained from sequencing and counting are well aligned.

In the diurnal cycle, all major prokaryotic phyla, except for the genus *Synechococcus* of the Cyanobacteria, exhibit a similar trend, with significantly higher abundance under light conditions compared to dark conditions. This suggests that the negative effects caused by sunlight radiation are not a major stressor directly influencing the abundance variations of these groups. Notably, the diurnal patterns of different prokaryotic groups appear to exhibit varying trends across different environments. Taking Proteobacteria as an example, according to He et al. (2024), Alphaproteobacteria and Gammaproteobacteria show no significant differences in their relative abundances between day and night [85]. In contrast, Olapade (2012) observes a clear diurnal pattern for these two classes, with trends completely opposite to those found in the present study [86]. In agreement with our findings, Chen et al. (2024) finds that the groups in Alphaproteobacteria are more abundant during the day [87]. The factors underlying these abundance patterns are also quite diverse. Members of the Gammaproteobacteria group possess genes responsible for light-harvesting pigments, which trigger cell division under sunlight [88,89,90]. Additionally, during our observation period, tidal heights during the day were generally higher than at night. The tides, which bring in available nutrients from the open sea, may also contribute to the observed changes in this group, given their well-documented opportunistic tendencies [91]. For Alphaproteobacteria, the majority of this group in our study belong to the SAR11 clade, a photoheterotrophic group containing proteorhodopsins, which can promote its growth under sunlight [92]. Furthermore, studies have shown that protistan grazing is an important factor regulating SAR11 cell abundance, with grazing being more active at night [93]. Actinobacteria contain rhodopsins (actinorhodopsins), a potential mechanism for supplementing energy through light absorption, and also exhibit inherent UV stress resistance [94,95]. A relatively large fraction of the available genomic data for marine Bacteroidetes also contains proteorhodopsins, and studies have shown that they exhibit higher resistance to solar radiation compared to other bacterioplankton groups [96,97,98]. Additionally, one study finds a significant positive correlation between the percentage of Bacteroidetes in the bacterial community and day length [99]. These and similar reasons may help explain why a high abundance of specific prokaryotic groups was observed during the day in our study.

The abundance variations of major microeukaryotic groups are primarily observed between the morning and evening. A study reveals that the microeukaryotic community exhibits a 24 h cycle in their beta diversity, with the largest community differences observed between a 12 h interval [87]. The sampling times of our morning and evening samples are exactly 12 h apart, which helps explain why the abundance variations of most groups are significant between morning and evening. The diurnal abundance patterns of these microeukaryotic groups are also often inconsistent in the records. For example, He et al. (2024) reports that Dinoflagellata abundance is higher at night than during the day, which can be explained by bursts of cell division at night [85]. Although both our and their studies contains night samples, the sampling time in our study (20:30) was 3–5 h earlier than their sampling window (23:00–02:00). However, the noon samples in our study (13:30) were relatively consistent with their daytime samples (13:00–14:00). Therefore, our night samples may not have captured this division peak, whereas the morning samples, which were collected closer to the peak of cell division, exhibit this increase in abundance. This highlights the importance of the sampling timepoint when conducting in situ observations of microbial communities, as a few hours’ difference can lead to completely different community outcomes. In addition, the raphid pennate diatoms, which make up the largest proportion after dinoflagellates, show significantly higher abundance at noon than in the morning, which may be attributed to their cell division during the day [100].

## 5. Conclusions

This study is the first to investigate the prokaryotic and microeukaryotic communities in the Zhubi lagoon using flow cytometry and high-throughput sequencing, and to explore diurnal microbial community patterns. The diurnal light cycle induces significant changes in dominant picocyanobacteria cell abundance, which further drives diurnal variations in microbial communities. Additionally, diurnal differences exhibited by various prokaryotic and microeukaryotic populations vary depending on habitats and sampling timepoints. Our findings enhance the understanding of the planktonic community in the Zhubi lagoon, aiding in the assessment of the ecological health status and effective conservation and restoration strategy development. Given our limited sampling location and time, further field investigations are needed.

## Figures and Tables

**Table 1:** ANOSIM-based hypothetical test of diurnal variations in beta diversity of prokaryotic and microeukaryotic community.

Static *R*	Prokaryote	Microeukaryote	*n*
Global	0.29 **	0.06	27
Time	0.53 ***	0.21 ***	9
Time at 1 m	0.53 **	0.41 *	3
Time at 10 m	0.32 *	0.13	3
Time at 20 m	0.51 **	−0.004	3
Depth	−0.05	−0.05	9
Depth in the evening (dark)	−0.16	−0.11	3
Depth in the morning (low light intensity)	−0.28	−0.17	3
Depth at noon (high light intensity)	−0.24	−0.15	3

The significant results are highlighted in bold. * *p* < 0.05, ** *p* < 0.01, *** *p* < 0.001.

## Data Availability

Raw sequence data (27 samples) generated in this study have been deposited in the Sequence Read Archive (SRA) database of the National Center for Biotechnology Information with the project number PRJNA1219476. The data that support the findings of this study are available from the corresponding author upon reasonable request.

## Supplementary Materials

The following supporting information can be downloaded at: https://www.mdpi.com/article/10.3390/microorganisms13040727/s1, Figure S1: Tidal height diagram during the sampling period; Figure S2: The variation of environmental variables between different times and depths; Figure S3: The intensity and attenuation of PAR in depths of 1 to 20 m at different sampling times; Figure S4: Comparison of picoplankton cell abundance across different depths; Figure S5: UPGMA clustering and phylum-level relative abundance composition of prokaryotic and microeukaryotic communities for each sample; Figure S6: Standardized direct and indirect mean effects of light, *Synechococcus*, and *Prochlorococcus* on microbial diversity; Table S1: Summary information for the sampling; Table S2: Stepwise regression analysis for predicting cell numbers of picophytoplankton from environmental variables; Table S3: Comparisons of alpha diversity indices at different depths in prokaryotic and microeukaryotic communities using the Wilcoxon rank sum test; Table S4: Stepwise regression analysis for predicting alpha diversity indices of prokaryotic and microeukaryotic community from environmental and biotic variables; Table S5: Spearman correlation between environmental distance and the dissimilarity of prokaryotic and microeukaryotic communities assessed using Mantel test.
